# Do Routine Preoperative and Intraoperative Urine Cultures Benefit Pediatric Vesicoureteral Reflux Surgery?

**DOI:** 10.1155/2017/3197869

**Published:** 2017-04-11

**Authors:** Daniel R. Hettel, Bradley C. Gill, Audrey C. Rhee

**Affiliations:** ^1^Lerner College of Medicine, Education Institute, Cleveland Clinic, Cleveland, OH, USA; ^2^Department of Urology, Glickman Urological and Kidney Institute, Cleveland Clinic, Cleveland, OH, USA; ^3^Children's Hospital and Pediatric Institute, Cleveland Clinic, Cleveland, OH, USA

## Abstract

*Objective*. To determine if routine preoperative and intraoperative urine cultures (UCx) are necessary in pediatric vesicoureteral (VUR) reflux surgery by identifying their association with each other, preoperative symptoms, and surgical outcomes.* Materials and Methods*. A retrospective review of patients undergoing ureteral reimplant(s) for primary VUR at a tertiary academic medical center between years 2000 and 2014 was done. Preoperative UCx were defined as those within 30 days before surgery. A positive culture was defined as >50,000 colony forming units of a single organism.* Results*. A total of 185 patients were identified and 87/185 (47.0%) met inclusion criteria. Of those, 39/87 (45%) completed a preoperative UCx. Only 3/39 (8%) preoperative cultures returned positive, and all of those patients were preoperatively symptomatic. No preoperatively asymptomatic patients had positive preoperative cultures. Intraoperative cultures were obtained in 21/87 (24.1%) patients; all were negative. No associations were found between preoperative culture results and intraoperative cultures or between culture result and postoperative complications.* Conclusions*. In asymptomatic patients, no associations were found between the completion of a preoperative or intraoperative UCx and surgical outcomes, suggesting that not all patients may require preoperative screening. Children presenting with symptoms of urinary tract infection (UTI) prior to ureteral reimplantation may benefit from preoperative UCx.

## 1. Introduction

Vesicoureteral reflux (VUR) is defined as the retrograde passage of urine from the bladder into the upper urinary tract [1]. VUR occurs in approximately 1% of children [2] and often presents at a young age as a urinary tract infection (UTI) [3]. Renal scarring is a long-term complication of recurrent UTI secondary to untreated VUR [1] and can lead to childhood hypertension, impaired renal and somatic growth, and, rarely, end-stage renal disease [1]. These consequences are often seen after years of recurrent UTI [4]. The effects of benign urine reflux, or retrograde passage of uninfected urine from the bladder to the kidney, have not been definitively identified [1]. As such, the prevention of recurrent UTI and preservation of kidney function are the overall goals of VUR treatment [5].

Management of VUR consists of surgical and nonsurgical approaches [6]. Open surgical repair is considered the gold standard and preferred by many pediatric urologists, with a 95–98% resolution of reflux and few complications [7, 8]. However, operative intervention carries risks [9], especially when an active UTI is present (i.e., sepsis and/or wound infection) [10]. To prevent this, many surgeons routinely obtain a urine culture (UCx) prior to surgery to confirm a sterile urinary tract and some surgeons obtain an intraoperative UCx to further prove this.

The utility of preoperative UCx prior to open reconstruction for treating VUR has not been investigated. The goal of this study was to retrospectively examine the relationships between preoperative UCx results and surgical outcomes, preoperative UCx results and intraoperative UCx results, and preoperative UCx results and patient presentation. The hypothesis of this study is that preoperative UCx results are not associated with postoperative complications, intraoperative UCx add little useful clinical information to practice, and patients requiring a preoperative UCx are readily clinically identifiable.

## 2. Methods

Institutional review board approval was obtained for a retrospective review of patients who underwent open surgical reconstruction for VUR between 2000 and 2014 at a large tertiary-care institution. Manual extraction of data from the electronic medical record (Table 1) included patient age at surgery, grade of VUR based upon the international reflux committee grading system [11], preoperative signs or symptoms of UTI, preoperative UCx, intraoperative UCx, postoperative complications, and UTI(s) within the year after surgery. Without a clear evidence-based definition of a positive preoperative UCx, UTI was defined as greater than 50,000 colony forming units of a single organism [12–14]. Preoperative UCx was defined as a specimen obtained within the 30 days before surgery. All urine specimens were collected via catheterization or a mid-stream clean catch. Multidrug resistance was defined as an organism resistant to one or more classes of antibiotics [15]. Preoperative signs and symptoms of UTI included fever, dysuria, cloudy urine, malodorous urine, or primary caregiver report of the “typical presentation” of UTI in that specific patient. Preoperative management, including antibiotic prophylaxis, varied per surgeon preference. Indications for surgery included but were not limited to breakthrough febrile UTI with antibiotic prophylaxis, worsening reflux on follow-up VCUG, and patient/provider preference. Postoperative complications included wound infection, wound breakdown, abnormal bleeding, delayed healing, ureteric obstruction, and urine leak. Postoperative management varied per surgeon preference, but all patients did undergo postoperative US within 6 months of surgery, as a repeat VCUG is not standard at this institution. The reimplant technique varied between intravesical (Cohen cross-trigonal) and extravesical (Table 1). All patients received antibiotics, typically cefazolin 50 mg/kg/dose, prior to incision.

Inclusion criteria were complete preoperative and perioperative medical records. Patients with otherwise complex and potentially confounding urologic conditions were excluded, including neurogenic bladder, ureterocele, posterior urethral valves, prune belly syndrome, ureteropelvic junction obstruction, or nephrolithiasis. Data analysis consisted of descriptive statistics and association testing using Fisher's exact test, as appropriate. Patients with incomplete data were included in analyses for which data was available.

## 3. Results

### 3.1. Preoperative Urine Cultures

**Figure 1:**
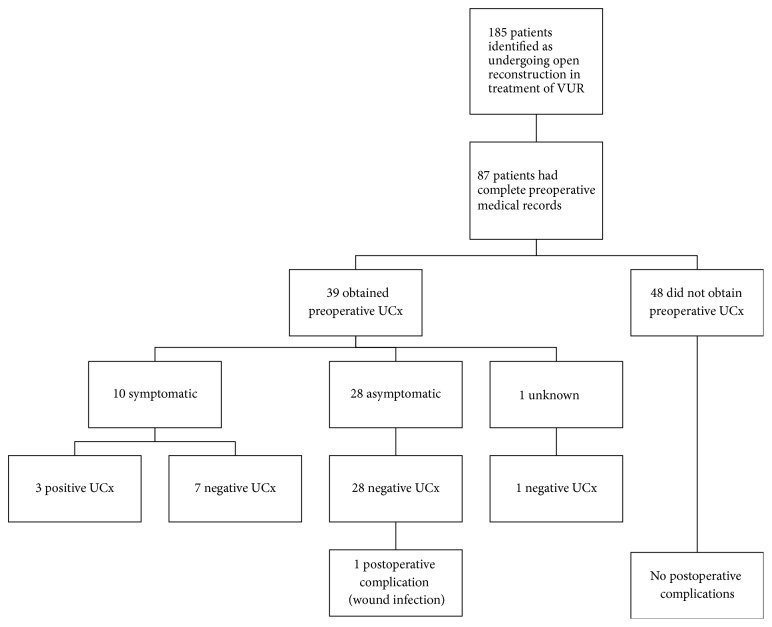
Preoperative urine culture results and postoperative outcomes.

A total of 185 patients underwent open reconstruction for VUR and 87 (47%) had complete preoperative medical records (Table 1). Manual data review found 39/87 (45%) completed a preoperative UCx, and of those, 10/39 (26%) patients were symptomatic: 6/10 (60%) experienced fever, 2/10 (20%) had malodorous urine, and 2/10 (20%) exhibited their “typical symptoms” per primary caregiver report. Only 3/10 (30%) symptomatic patients returned positive UCx, of which 2 had malodorous urine and the remaining 1 had a typical symptomatic presentation (Figure 1). All 3 were treated with oral antibiotics prior to surgery. Positive preoperative UCx were significantly (*p* = 0.01) associated with a symptomatic presentation.

All 10 preoperatively symptomatic patients with UCx were followed up for at least 1 year. None experienced postoperative complications. The 3 patients with positive preoperative UCx had no recurrent UTIs postoperatively, while 2/7 (29%) symptomatic patients with negative preoperative cultures had at least 1 UTI documented within the first postoperative year. A positive preoperative UCx was not associated with UTI recurrence during the first postoperative year (*p* = 1.00), nor was a symptomatic preoperative presentation (*p* = 1.00).

Of the 28 asymptomatic patients with UCx, 3/28 (11%) were lost to follow-up within the first postoperative year. One experienced a postoperative wound infection, which was initially treated with oral antibiotics prior to eventual wound debridement, which returned sterile wound cultures. Within the remaining 24 asymptomatic patients with UCx, recurrent UTI was noted in 5/24 (21%) patients within the first postoperative year.

No significant difference was found between preoperatively symptomatic and asymptomatic patients with regard to postoperative complications (*p* = 1.00) or recurrent UTI (*p* = 0.65). Otherwise, one patient with UCx but unknown symptom status at presentation returned a negative preoperative urine culture and experienced no postoperative complications prior to being lost to follow-up 7 months postoperatively.

Complete preoperative records were collected for 48 patients without preoperative UCx. No postoperative complications were noted in this group. Follow-up data for at least the first postoperative year was available for 40/48 (83%) of these patients, of which 7/40 (17%) experienced UTI recurrence. There was no association between postoperative complications (*p* = 0.45) or recurrent UTI (*p* = 1.00) and foregoing a preoperative UCx (Table 2).

### 3.2. Intraoperative Urine Culture

Intraoperative UCx were collected in 21 patients with complete medical records. All intraoperative UCx were negative. No postoperative complications were noted in patients with intraoperative UCx completion. Recurrent UTI was noted in 4 cases and all 4 patients were asymptomatic prior to surgery, but only 1 completed a preoperative UCx, which returned negative. There was no association between intraoperative UCx results and postoperative complications (*p* = 1.00) or recurrent UTI (*p* = 0.498).

### 3.3. Drug-Resistant Uropathogens

Of all 87 patients, 44/87 (51%) had a history of MDR uropathogens. Of the 39 patients with preoperative UCx, 21 (54%) had a history of MDR UTI, including all 3 patients with positive preoperative UCx, despite none of the MDR microorganisms being detected. No association between MDR uropathogen history and positive preoperative UCx (*p* = 0.24) existed (Table 2). Of the 48 patients that did not complete a preoperative UCx, 23/48 (48%) had a history of MDR uropathogens. Overall, no association between a history of MDR uropathogens and recurrent postoperative UTI (*p* = 0.77) existed.

## 4. Discussion

Preoperative UCx is not currently addressed in American Urological Association practice guidelines [5] and obtaining preoperative UCx is at surgeon discretion. Many pediatric patients are asked to provide a UCx within days of surgery. As a practice predominantly in pediatric urology [16, 17], this requires parents to allocate time and resources for obtaining UCx. To the knowledge of the authors, it is unknown if these UCx have an effect on surgical outcomes. This study found that the patients who may benefit from screening UCx can likely be identified by clinical presentation. Specifically, all asymptomatic patients in this series had negative preoperative UCx, while positive preoperative UCx were found in only symptomatic patients (Figure 1). Furthermore, all patients returning positive preoperative cultures also had a history of MDR UTI. Overall, these data suggest that preoperative UCx can likely be safely limited to symptomatic patients.

No associations between preoperative UCx results and postoperative complications were noted in this study. Furthermore, no associations were found between postoperative recurrent UTI and any of the preoperative or intraoperative measures assessed in this study. The single postoperative complication occurred in a preoperatively asymptomatic patient with negative UCx. As no patients foregoing preoperative UCx experienced postoperative complications, it can be argued that factors besides bacteriuria could underlie the risk of postoperative complications.

A relative paucity of literature describing postoperative complications in VUR surgery exists. That, which is available, lacks consistent classifications and definitions of complications [8, 18–20]. The European arm of the International Reflux Study in Children found ureteral obstruction as the most common complication [20], whereas a more recent study by Marchini and colleagues indicated significant bladder spasm and hematuria were the most common complications [18]. Neither the study presented in this manuscript, nor those mentioned here, utilized the same definition of complication in open VUR surgery. As a whole, this hinders comparisons between studies.

In adults, asymptomatic bacteriuria, detected prior to prosthetic joint replacement, is a risk factor for implant infection [21]. Sousa et al. found the presence of asymptomatic bacteriuria before joint replacement imparted an odds ratio of 3.67 (CI 1.67–6.27; *p* < 0.05) for postoperative joint infection relative to a sterile urine culture. Urine-directed treatment before joint replacement was not mandatory or randomized, and those treated for asymptomatic bacteriuria experienced similar prosthetic joint infection rates as those who were not treated. The authors concluded that asymptomatic bacteriuria does not require preoperative treatment, which could suggest ubiquitous preoperative urine cultures are unnecessary in this population. These results resemble those found in the current study, in that asymptomatic children who forwent preoperative UCx experienced no increases in postoperative complication rates or recurrent UTI compared to asymptomatic children who did receive urine cultures (Table 2). Although the demographics of these study samples differ, their conclusions are similar and suggest factors other than preoperative bacteriuria affect postoperative complications and recurrent UTIs.

The practice of obtaining intraoperative UCx is often surgeon and center specific, and its benefit is unknown at this time. A study investigating positive intraoperative UCx obtained during open reconstruction in treatment of VUR found that positive cultures returned in only 6 (2.56%) of 234 patients over 2 years [22]. This led the authors to conclude that routine intraoperative UCx are of minimal benefit and should only be obtained in the presence of positive preoperative UCx or abnormal cystoscopic findings. The current study is in agreement with these findings, as no positive intraoperative UCx returned, and hence no associations between preoperative UCx results and intraoperative UCx results or intraoperative UCx results and outcomes were present. Combined, these studies show that intraoperative UCx add little useful clinical data to guide practice. In some cases, such as finding frankly purulent urine upon entering the bladder, UCx is indicated. In these instances, obtaining a UCx is important and likely to impact patient care.

The study presented herein examines how outcomes of surgical VUR correction are impacted by preoperative UCx results. A drawback of this study is the relatively small sample size, which may account for the lack of positive intraoperative UCx observed. Likewise, the lack of complete preoperative medical records for all patients presents another challenge often inherent to retrospective reviews, which further limited sample size and restricted population heterogeneity. A third limitation is that some patients were lost to follow-up. Although it is unlikely that those patients experienced postoperative complications without reporting back to the surgeon, it is always possible that a recurrent UTI or other issue may have been treated at another institution. With that, further characterization of bowel and bladder dysfunction in the study sample could strengthen its findings, as multiple studies have indicated that defecatory issues impact rates of UTI recurrence and VUR resolution [23–25]. Lastly, it should be understood that the absence of a preoperative UCx does not necessarily mean cultures were forgone, as it is possible that they may have been performed somewhere other than the tertiary-care center in which this study is based. However, the vast majority of the patients included in this study received primary and urologic care from the health system involved, which is documented in a comprehensive electronic medical record.

The data presented in this manuscript demonstrate no associations between preoperative or intraoperative UCx results and postoperative outcomes. Patients presenting with UTI symptoms were significantly more likely to have positive preoperative UCx, suggesting that, in a population similar to the one under review, screening can be limited to symptomatic patients only. This is further supported by the fact that no preoperatively asymptomatic patients had positive urine cultures. These findings are likely related to the successful treatment of identified preoperative UTI and sufficient perioperative antibiotic administration throughout the entire sample. Lastly, this study, and the published literature, clearly shows the utility of intraoperative UCx is negligible, suggesting it should not be performed without indication. Overall, the findings in this study provide an impetus to pursue further investigation of this issue in a prospective fashion.

## 5. Conclusion

For patients undergoing ureteral reimplantation for vesicoureteral reflux, the results of preoperative and intraoperative urine cultures were unrelated to postoperative outcomes and urinary tract infection recurrence. As positive preoperative urine cultures were significantly more likely to occur in symptomatic than asymptomatic patients, foregoing preoperative urine cultures may not be a contraindication to surgery in asymptomatic patients. Intraoperative urine cultures added no information that influenced clinical management and ubiquitous screening should be abandoned in light of adding unnecessary cost. Further prospective study may further elucidate the role of preoperative UCx in pediatric urology.

## Figures and Tables

**Table 1:** Patient demographics of all 185 patients found to have ureteral reimplantation between 2000 and 2014 and the corresponding 87 patients with complete electronic medical records.

Parameter	Total study population		Patients with complete EMR	
	Female	Male	Female	Male
Number of participants (%)	157 (85%)	28 (15%)	77 (89%)	10 (11%)
Median age at surgery [Inner quartile range]	4.00 [2–5]	3.00 [1–5]	3.00 [1.5–5]	4.00 [1.75–6.25]

Laterality of VUR: *N* (%)				
(i) Left	36 (19%)	8 (4.0%)	14 (18%)	4 (40%)
(ii) Right	24 (13%)	5 (3.0%)	6 (8%)	1 (10%)
(iii) Bilateral	94 (51%)	13 (7.0%)	56 (73%)	4 (40%)
(iv) Unknown	3 (2.0%)	2 (1.0%)	1 (1%)	1 (10%)

Severity of VUR: *N* (%)				
(i) I	2 (1.0%)	0 (0.0%)	0 (0%)	0 (0%)
(ii) II	33 (18%)	4 (2.0%)	12 (16%)	1 (10%)
(iii) III	66 (36%)	4 (2.0%)	31 (40%)	1 (10%)
(iv) IV	38 (21%)	8 (4.0%)	28 (36%)	4 (40%)
(v) V	3 (2.0%)	10 (5.0%)	1 (1%)	3 (30%)
(vi) Unknown	15 (8.0%)	2 (1.0%)	5 (7%)	1 (10%)

Collecting system: *N* (%)				
(i) Single	130 (70%)	21 (11%)	60 (78%)	9 (90%)
(ii) Duplicate	25 (14%)	5 (3.0%)	16 (21%)	1 (10%)
(iii) Unknown	2 (1.0%)	2 (1.0%)	1 (1%)	0 (0%)

Patient presentation: *N* (%)				
All patients				
(i) UTI	129 (70%)	11 (6.0%)	69 (90%)	6 (60%)
(ii) Prenatal screening	7 (4.0%)	11 (6.0%)	4 (5%)	3 (30%)
(iii) Sibling screening	1 (0.5%)	2 (1.0%)	1 (1%)	0 (0%)
(iv) Unknown	20 (11%)	4 (2.0%)	3 (4%)	1 (10%)
Preoperative UCx patients				
(i) UTI	31 (79%)	4 (10%)	31 (79%)	4 (10%)
(ii) Prenatal screening	1 (3.0%)	2 (5.0%)	1 (3.0%)	2 (5.0%)
(iii) Sibling screening	1 (3.0%)	0 (0.0%)	1 (3.0%)	0 (0.0%)
(iv) Unknown	0 (0.0%)	0 (0.0%)	0 (0.0%)	0 (0.0%)

Surgical technique				
Cohen Cross-trigonal	97 (62%)	16 (57%)	56 (73%)	8 (80%)
Extravesical reimplant	52 (33%)	10 (36%)	16 (21%)	1 (10%)
Other	8 (5.0%)	2 (7.0%)	5 (6%)	1 (10%)

**Table 2:** Association testing between patient groups and outcomes or characteristics.

	Number of patients	*p* value
*Positive preoperative UCx*	3	—
(i) Preoperatively symptomatic	3	*p* = 0.01
(ii) Preoperatively asymptomatic	0	

*Postoperative complications*	1	—
(i) Positive preoperative UCx	0	*p* = 1.00
(ii) Negative preoperative UCx	1	
(i) Preoperatively symptomatic	0	*p* = 1.00
(ii) Preoperatively asymptomatic	0	
(i) No preoperative UCx obtained	0	*p* = 0.45
(ii) Preoperative UCx obtained	1	

*UTI within 1 year of surgery*	14	—
(i) Positive preoperative UCx	0	*p* = 1.00
(ii) Negative preoperative UCx	5	
(i) Preoperatively symptomatic	5	*p* = 0.65
(ii) Preoperatively asymptomatic	9	
(i) No preoperative UCx obtained	8	*p* = 1.00
(ii) Preoperative UCx obtained	5	

*History of MDR*	44	—
(i) Positive preoperative UCx	3	*p* = 0.24
(ii) Negative preoperative UCx	18	
(i) Preoperatively symptomatic	7	*p* = 0.08
(ii) Preoperatively asymptomatic	20	
(i) No preoperative UCx obtained	23	*p* = 0.67
(ii) Preoperative UCx obtained	21	
